# A clinical drug-drug interaction assessment using physiologically based pharmacokinetic modeling: a case study with chloroquine and colchicine

**DOI:** 10.1590/1414-431X2026e15326

**Published:** 2026-07-10

**Authors:** T.R. Maciel, F.E.G. Teixeira, I.C. Bitencourt, C.O. Pacheco, S.E. Haas

**Affiliations:** 1 Laboratório de Farmacologia e Farmacometria, Universidade Federal do Pampa, Uruguaiana, RS, Brasil; 2 Programa de Pós-Graduação em Ciências Farmacêuticas, Universidade Federal de Santa Maria, Santa Maria, RS, Brasil

**Keywords:** Data-driven modeling, PBPK, DDI, Chloroquine, Colchicine, Malaria

## Abstract

The combination of drugs for malaria treatment holds promise, although the potential for drug-drug interactions remains insufficiently explored in novel therapeutic combinations. This study aims to assess these interactions using physiologically based pharmacokinetic modeling, supported by data-driven parameter optimization, as a step towards the preclinical development of a formulation containing chloroquine and colchicine. Given that both compounds share metabolic pathways involving CYP3A4 and CYP2D6, we developed individual and population models using a middle-out strategy in PK-Sim^®^, an open-source software, and validated these models by comparing predicted and observed pharmacokinetic parameters. Simulations evaluated competitive inhibition between the compounds. The results indicated no significant changes in systemic exposure, with the area under the curve and maximum concentration values remaining consistent between single and combined administration. Our findings suggest that the proposed modeling is a powerful tool for predicting pharmacokinetic interactions during the preformulation stage, offering mechanistic insight and supporting rational decision-making prior to *in vivo* studies. The absence of significant drug-drug interactions between chloroquine and colchicine reinforces the feasibility of advancing this combination in future therapeutic development.

## Introduction

Recent advances in computational modeling and data science have contributed to significant progress in the early phases of drug development, particularly in predicting pharmacokinetic interactions before clinical trials begin (1). In this context, physiologically based pharmacokinetic (PBPK) modeling has emerged as a key tool, providing a mechanistic framework to simulate drug absorption, distribution, metabolism, and excretion using biological and physicochemical data (2). PBPK modeling has been increasingly combined with data-driven optimization strategies to refine parameter estimation and to support the simulation of complex drug-drug interactions (DDIs), especially in preclinical scenarios where experimental evidence is limited. These approaches are especially valuable in neglected or challenging therapeutic areas such as malaria, where ethical, logistical, and biological constraints often restrict traditional experimental designs (1).

Despite significant efforts and advancements outlined by the World Health Organization's global strategy against malaria, progress has stagnated. In 2023, an estimated 263 million cases of malaria and 597,000 related deaths were reported worldwide, marking an increase of 11 million cases from the previous year (3).

Combination therapy remains a principal strategy for treating malaria, primarily due to the persistence of parasite resistance, the main challenge in combating the disease (4). Artemisinin-based dual combination therapy has long been recommended by the World Health Organization for severe malaria, although treatment-resistant strains have begun to emerge (5), leading to artemisinin-based triple combinations being studied and tested (6).

Combining drugs to treat malaria introduces concerns about DDIs, which remain underexplored. Moreover, new drug associations are continuously being identified (7), highlighting the evaluation of such interactions as limited, particularly in the early developmental stages. This limitation signifies a considerable gap in knowledge crucial for the rational design of combination therapies (2).

Specifically in malaria research, PBPK modeling has been instrumental in examining antimalarial drug exposure and refining dosing strategies, especially in unique populations, such as pregnant women and children (8,9). These models also facilitate the early assessment of potential DDIs, which is vital given the frequent polypharmacy in malaria treatment, including coadministration of antibiotics or antivirals (7,10).

Our preceding study introduced an innovative nanocapsule formulation of chloroquine (CQ) and colchicine (CC) for malaria treatment, assessing physicochemical compatibility and potential drug and excipient interactions *in vitro*. This groundwork enabled the development of a nanocarrier system for the concurrent delivery of both agents (11). However, physiological interaction still requires evaluation.

Pharmacokinetically, CQ is characterized by a high volume of distribution (Vd), prolonged terminal half-life, and primary hepatic metabolism via cytochrome P450 (CYP) 2C8, CYP3A4, and CYP2D6 (12). Despite literature describing enantioselective disposition, our model utilized the racemic mixture, the clinically relevant form (13). On the other hand, CC is a drug with a narrow therapeutic index, extensively metabolized by CYP3A4 and transported by P-glycoprotein (P-gp), prone to pharmacokinetic variability and DDI (14,15). Considering these characteristics and the common coadministration of antimalarial and adjunctive drugs, our modeling approach allows a mechanistic evaluation of systemic exposure and potential interactions at the preformulation stage (16). With this pharmacokinetic foundation and established formulation, an *in-silico* method is pivotal in predicting potential metabolic interactions and optimizing combination therapy.

In this study, we applied PBPK modeling, supported by data-driven techniques, to forecast potential DDIs between CQ and CC, two repurposed compounds under study for experimental antimalarial combination therapy. Due to both drugs being metabolized by CYP3A4 and CYP2D6, there is a concern for metabolic competition. By simulating the pharmacokinetic profiles of these drugs both individually and in combination, we aim to assess *in silico* pharmacokinetic interactions, contributing to the safer and more effective design of combination therapies targeting malaria in the preclinical stage.

## Material and Methods

### Overview of the modeling strategy

**Figure 1:**
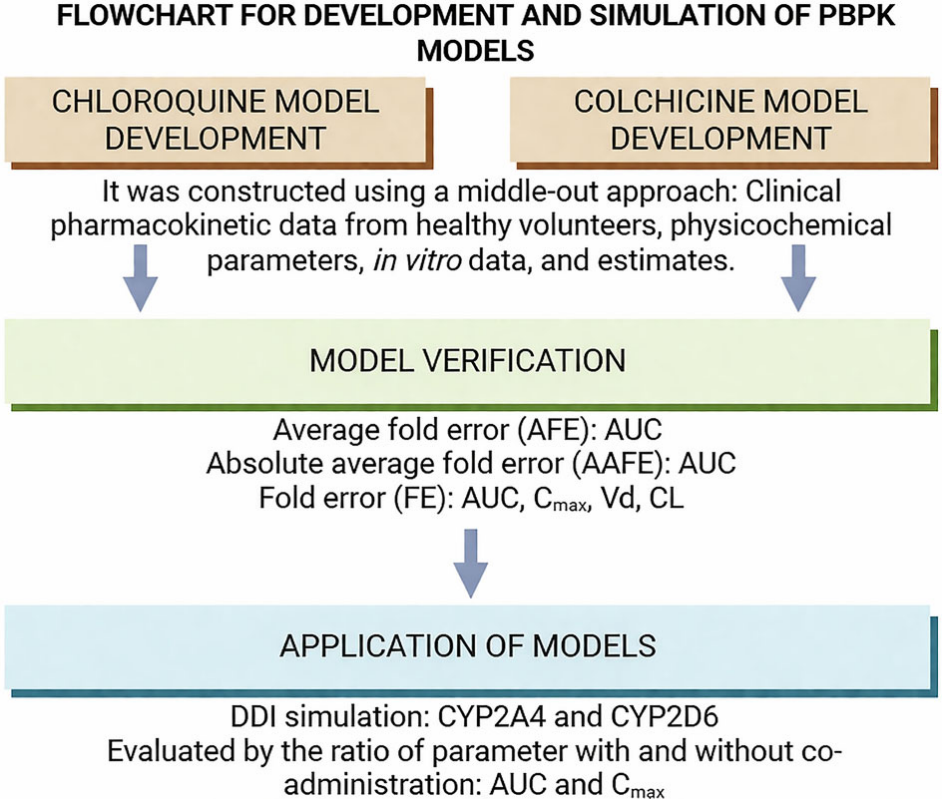
Flowchart depicting the study design. C_max_: maximum concentration; Vd: volume of distribution; CL: clearance; DDI: drug-drug interactions; AUC: area under the curve.

The PBPK models for CQ and CC were developed utilizing PK-Sim 12.0, an open-source software package. The models employed data from the standard European virtual population software, incorporating clinical pharmacokinetic data from healthy volunteers, physicochemical parameters, *in vitro* data, and estimates for CQ and CC. The model development process was divided into three stages: individual model development, model evaluation, and model simulation/application (Figure 1).

### Model development

The models for CQ and CC were constructed employing a middle-out approach that integrates bottom-up and top-down techniques (17). This involved the collection of data on physicochemical characteristics, absorption, distribution, metabolism, and excretion processes, as well as clinical studies involving intravenous and oral administration in healthy subjects for both CQ and CC, deriving from an extensive literature review (18-23). All referenced data were sourced from clinical or preclinical studies (9,12,24).

The development of both models was predicated on a protocol of single-dose oral administration, assuming immediate release, and was based on a healthy European male, aged 30 years, weighing 74 kg, with a height of 172.40 cm and a body mass index of 22.88 kg/m^2^. Other physiological parameters were aligned with those provided by simulation software. The intracellular partition coefficient for plasma was estimated using Rogers and Rowland's method, with cell permeability calculated via the standard PK-Sim^®^ method.

Expression data for metabolizing enzymes and transport proteins were integrated into the models using the PK-Sim^®^ Expression Database. This database offers organ-specific expression levels derived from pooled RT-PCR datasets and proteomic data (25). For CQ, the enzymes CYP1A2, CYP2C19, CYP2C8, CYP2D6, CYP3A4, and CYP3A5 were included. For CC, the enzymes CYP2D6 and CYP3A4, along with the transport protein ABCB1 (P-gp), were incorporated (8,12).

For parameters that could not be directly retrieved from the literature or the PK-Sim^®^ databases, the PK-Sim^®^ parameter identification tool (parameter identification module) was used with a Monte Carlo optimization algorithm to estimate values based on observed concentration-time data. For CQ, the primary dataset utilized was from Gustafsson et al. (19), which included single-dose regimens of 300 mg administered intravenously and orally to healthy subjects. Additional data were sourced from Neuvonen et al. (20), involving a 500-mg oral dose; Pukrittayakamee et al. (21), with a 600-mg oral dose, and Walker et al. (23), also with a 600-mg oral regimen. For CC, model calibration relied on the study by Ferron et al. (18), which investigated an intravenously administered dose of 0.5 mg, and Thomas et al. (22), with 0.5 mg oral administration in healthy volunteers. Complementary data were included from Terkeltaub et al. (14), who administered a 0.6-mg oral dose, and Thomas et al. (22), who evaluated oral doses of 0.5 and 1.5 mg.

These datasets were digitized and served as targets for model fitting, with studies selected for their well-characterized pharmacokinetic profiles, detailed dosing regimens, participant demographics, administration routes, and sampling schedules (Table 1). The modeling approach adhered to a middle-out strategy, seamlessly integrating bottom-up elements (e.g., physicochemical and *in vitro* parameters) with top-down adjustments from clinical data to achieve physiologically plausible and predictive models.

**Table 1:** Studies used to develop and evaluate the physiologically based pharmacokinetic model.

Drug	Dose	Route	Reference
Chloroquine	300 mg	Oral solution	Gustafsson et al. 19
	300 mg	Oral tablet	Gustafsson et al. 19
	300 mg	Intravenous	Gustafsson et al. 19
	500 mg	Oral solution	Neuvonen et al. 20
	600 mg	Oral solution	Pukrittayakamee et al. 21
	600 mg	Oral solution	Walker et al. 23
Colchicine	0.5 mg	Intravenous	Ferron et al. 18
	1.0 mg	Oral solution	Ferron et al. 18
	1.0 mg	Oral tablet	Ferron et al. 18
	0.5 mg	Oral solution	Thomas et al. 22
	1.5 mg	Oral solution	Thomas et al. 22
	0.6 mg	Oral solution	Terkeltaub et al. 14

### Model evaluation

To quantitatively predict the variability in simulated plasma concentration-time profiles, virtual populations comprising 100 individuals aged 20-50 years were generated based on the previously developed single-individual model. The physiological parameters for this age range were maintained constant and calculated using specialized software. Simulations of these populations were generated, and their outcomes were then compared with observed data.

The models' performances were evaluated by comparing the simulated concentration-time profiles and the area under the concentration-time curve (AUC) with those observed. To assess the descriptive and predictive accuracy of each model, three metrics were employed: the average fold error (AFE) (Equation 1), the absolute average fold error (AAFE) (Equation 2), and the fold error (FE) (Equation 3) for the AUC, maximum concentration (C_max_), Vd, and clearance (CL) parameters. Acceptable values for AFE ranged from 0 to 2, for AAFE from 1 to 2 (26), and for FE, permissible values were stipulated to be between 0.5 and 2 (27).

$$AFE=10^{\frac{1}{n}\sum \log\left(\frac{PRED_{t}}{OBS_{t}}\right)}$$ (Eq. 1)

$$AAFE=10^{\frac{1}{n}\sum \left|\log\left(\frac{PRED_{t}}{OBS_{t}}\right)\right|}$$ (Eq. 2)

$$FE=\frac{\text{Observed}\;\text{parameter}}{\text{Predicted}\;\text{parameter}}$$ (Eq. 3)

where AFE is the average fold error for all ASC_0-inf_ predictions generated by the model, calculated from the logarithmic mean; PRED_t_ is the simulated concentration at time t; OBS_t_ is the observed concentration at the same time; and n is the number of evaluated time points. AFE measures predictive ability, indicating whether the model tends to over- or under-predict its target values.

In contrast, AAFE represents the absolute average fold error and is computed from the mean of the absolute logarithmic differences between predicted and observed concentrations. By incorporating the absolute value, AAFE equally accounts for both over- and under-predictions, providing a measure of overall accuracy regardless of direction.

Finally, FE quantifies the discrepancy between observed and predicted parameters (AUC, C_max_, CL, and Vd).

### PBPK model application and DDI simulations

**Figure 2:**
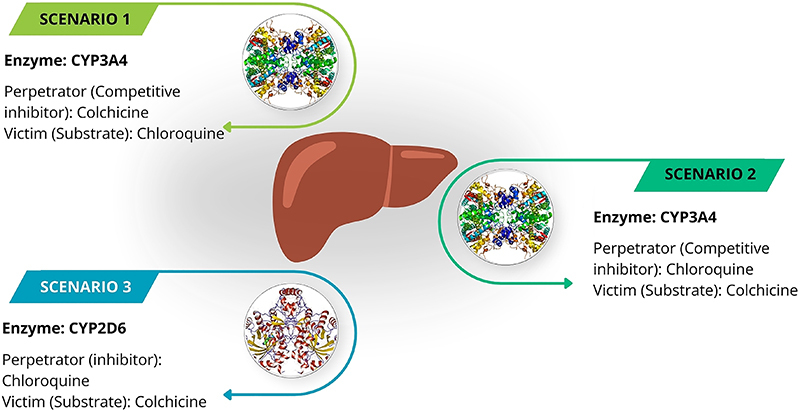
Schematic representation of the drug-drug interaction (DDI) simulations evaluated in this study.

The final CQ and CC models were integrated to assess DDI through the involvement of CYP3A4 and CYP2D6 substrates over 96 h for CQ and 48 h for CC following a single oral dose. For CYP3A4 substrates, simulations aimed to delineate the impact of CQ and CC through concurrent competitive inhibition of CYP3A4. In the case of CYP2D6, CQ was incorporated as an inhibitor with an inhibition constant (ki) of 3.15 µmol/L (12), and CC was analyzed as the affected substrate (Figure 2).

The DDI simulations were assessed by comparing plasma concentration-time profiles of the test drug alone *versus* those observed during concurrent administration. The primary metric employed was the AUC of DDI (Equation 4) to gauge the precision of each predicted interaction. According to the International Council for Harmonisation M12 guideline on drug interaction studies, a DDI is deemed not clinically significant if the AUC ratio between the co-administered and the single-drug scenarios falls within the no-effect boundaries of 0.80-1.25. Ratios deviating from this range may signify clinically meaningful interactions, necessitating further investigation (28).

$$Ratio\;DDI\;ASC_{0-\inf}=\frac{ASC_{0-\inf}\;\text{drug}\;\text{during}\;\text{co}-\text{administration}}{ASC_{0-\inf}\;\text{drug}\;\text{without}\;\text{co}-\text{administration}}$$ (Eq. 4)

## Results and Discussion

The PBPK approach was selected because it provides a mechanistic representation of absorption, distribution, metabolism, and elimination, integrating physiological and enzymatic parameters directly involved in potential DDI. This enables prospective evaluation of different clinical scenarios and prediction of changes in systemic exposure, which was central to the objectives of this study (1,2,16). In addition, alternative strategies, such as classical compartmental models or other commercial PBPK platforms, were considered (17,29). However, PK-Sim^®^ was chosen because it is open-source, widely validated, provides consolidated physiological libraries, and offers high reproducibility, making it particularly suitable for exploratory mechanistic CQ/CC interactions in the preclinical context (2,25).

### Model development and evaluation

The PBPK models were developed to optimize the dosing of CQ for treating COVID-19 (12) and the Zika virus during pregnancy (9). For the CC counterpart, however, no published PBPK models were identified. The added or estimated physicochemical parameters for both drugs and data from the literature of the model are detailed in Table 2.

**Table 2:** Physicochemical properties and pharmacokinetic parameters of chloroquine and colchicine used to develop the physiologically based pharmacokinetic models.

	Chloroquine			Colchicine	
	Data	Reference		Data	Reference
Molecular weight	319.87	Cui et al. 12		399.44 g/mol	DrugBank 40
PKa	8.40; 9.94	Redhi et al. 8		Neutral	DrugBank 40
Lipophilicity	1.77	Estimated		5.68	Estimated
Free fraction (%)	40.00	Estimated		75.48	Estimated
Solubility at pH 7	10.00 mg/mL	Estimated		0.24 mg/mL	Estimated
CYP1A2	Kcat 0.10 min^-1^	Estimated		-	-
CYP2C19	Kcat 1.83 min^-1^	Estimated		-	-
CYP2C8	Kcat 52.10 min^-1^	Redhi et al. 8		-	-
CYP2D6	Kcat 1.76 min^-1^	Estimated		Kcat 0.57 min^-1^	Estimated
CYP3A4	Kcat 166.50 min^-1^	Redhi et al. 8		Kcat 432.74 min^-1^	Estimated
CYP3A5	Kcat 41.56 min^-1^	Estimated		-	-
Renal clearance	-	-		TS 0.02 µmol/L/min	Estimated
CYP2D6 inhibition	3.15 uM/L	Cui et al. 12		-	-
P-gp (ABCB1)	-	-		Kcat 0.26 min^-1^	Estimated
Biliary clearance	-	-		CL 45.38 min^-1^	Estimated

TS: Tubular secretion; Kcat: turnover number; CL: clearance.

The development of the drugs' PBPK models utilized clinical data from healthy individuals, based on the understanding that CQ's pharmacokinetics are not significantly altered by disease (30). In the absence of CC data from malaria patients, all CC parameters were retained as initially defined.

**Figure 3:**
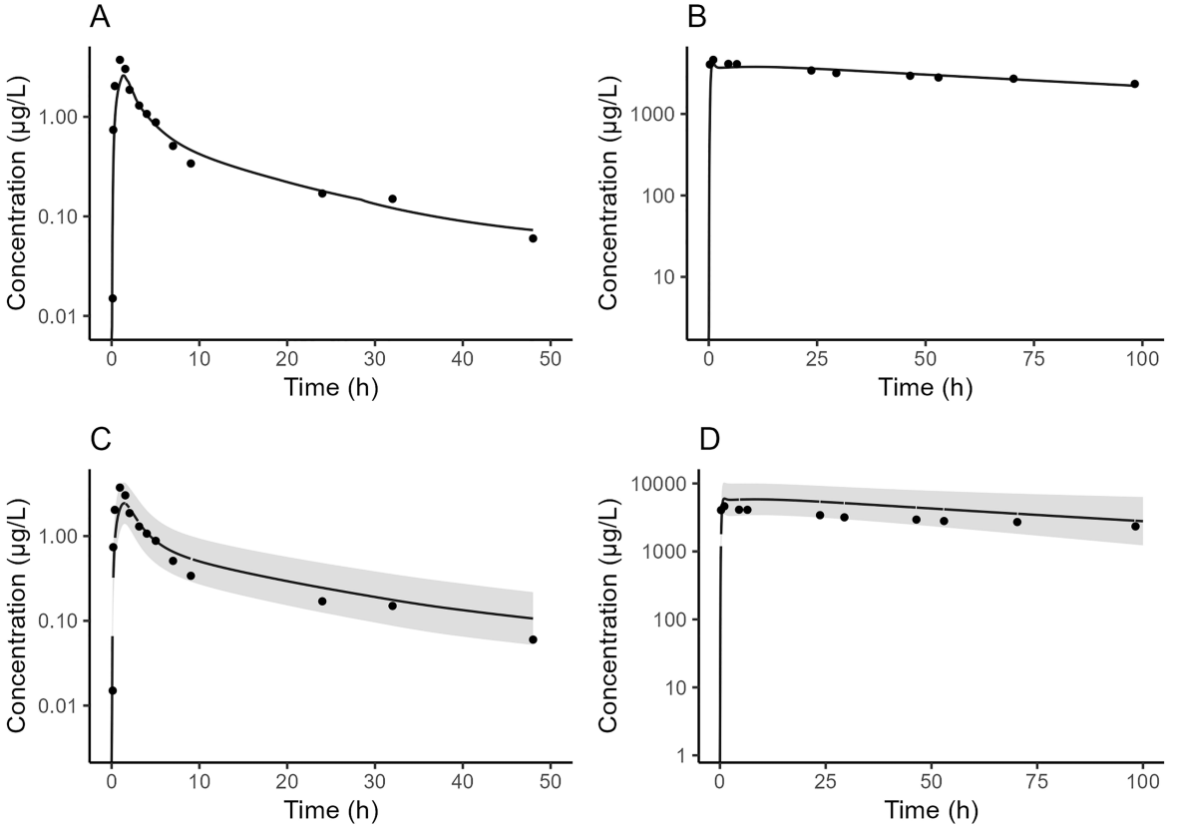
Concentration-time profiles for chloroquine (CQ) and colchicine (CC) after a single oral dose in healthy subjects and a virtual population (n=100). **A**, individual CC; **B**, individual CQ; **C**, population CC; **D**, population CQ. The solid lines represent the predicted mean concentration-time profile, the gray shaded area represents the geometric standard deviation, and the dots represent clinical data reported by Gustafsson et al. (19) for CQ (300 mg, oral solution) and Thomas et al. (22) for CC (0.5 mg, oral solution).

The PBPK model for CQ began with incorporating 300 mg of intravenous data (19) to refine the elimination and distribution parameters without the variability introduced by absorption. Following the satisfactory estimation of systemic CL and Vd, the model was expanded to include oral administration simulations (19) (Supplementary Figure S1). The finalized CQ model assumed a simple 300-mg oral dose in an immediate-release formulation (Figure 3). The CC model was similarly created, starting with 0.5 mg of intravenous data (18) to adjust the elimination kinetics (Supplementary Figure S1), and subsequently incorporating data from 1 mg of a single oral dose (22) (Figure 3). After establishing the fundamental structural parameters, further simulations were conducted using reported oral dosing regimens from multiple clinical studies (Table 1 and Supplementary Figures S2-S5), facilitating the models' evaluation and refinement under varied conditions.

The efficacy of both models was evaluated by comparing predicted and observed pharmacokinetic parameters (C_max_, Vd, CL, and AUC) and plasma concentration-time profiles (Figure 3). Simulations employed various oral and intravenous dosing regimens from the clinical studies listed in Table 3. The models' predictive accuracy was assessed with the AFE and AAFE for the AUC, and the FE for C_max_, CL, and Vd. The AFE values fell between 0.96 and 1.06, while AAFE values remained below 1.3, indicating high predictive precision. Moreover, the FE values for C_max_, Vd, and CL were within the accepted range of 0.5-2.0 for most simulations, confirming the reliability of the developed PBPK models in capturing the pharmacokinetics of CQ and CC across a range of dosing scenarios. This supported their use in further DDI simulations.

**Table 3:** Predicted and observed pharmacokinetic parameters for chloroquine and colchicine.

Dose, route (reference)	Parameters	Observed	Predicted	FE	AFE	AAFE
Chloroquine						
300 mg, *iv* 19	AUC (µg/L*h)	710,942.60	653,268.75	1.09	1.02	1.22
	C_max_ (µg/L)	7701.95	14,028.73	0.55	-	-
	Vd (L)	0.06	0.07	0.84	-	-
	CL (L/h)	0.00042	0.00046	0.92	-	-
300 mg, oral solution 19	AUC (µg/L*h)	841,854.40	646,371.01	1.30	1.02	1.49
	C_max_ (µg/L)	4,617.29	4,267.97	1.08	-	-
	Vd (L)	0.08	0.07	1.14	-	-
	CL (L/h)	0.00036	0.00046	0.78	-	-
300 mg, oral tablet 19	AUC (µg/L*h)	688,401.30	752,128.44	0.92	1.00	1.52
	C_max_ (µg/L)	4,821.05	5,890.78	0.82	-	-
	Vd (L)	58.90	46.00	1.29	-	-
	CL (L/h)	0.44	0.40	1.10	-	-
500 mg, oral solution 20	AUC (µg/L*h)	6,443.75	11,208.49	0.57	1.06	1.89
	C_max_ (µg/L)	61.95	39.58	1.57	-	-
	Vd (L)	3,657.54	2,290.00	1.60	-	-
	CL (L/h)	77.59	44.61	1.74	-	-
600 mg, oral solution 21	AUC (µg/L*h)	5,216.42	7,911.96	0.66	1.03	1.79
	C_max_ (µg/L)	250.14	247.08	1.01	-	-
	Vd (L)	2,695.13	4,183.49	0.64	-	-
	CL (L/h)	115.02	75.83	1.52	-	-
600 mg, oral solution 23	AUC (µg/L*h)	4,956.85	24,737.35	2.00	1.05	1.76
	C_max_ (µg/L)	465.07	795.17	0.59	-	-
	Vd (L)	1,261.43	982.73	1.28	-	-
	CL (L/h)	12.11	24.25	0.51	-	-
Colchicine						
0.5 mg, *iv* 18	AUC (µg/L*h)	300.65	266.34	1.13	1.03	1.44
	C_max_ (µg/L)	10.85	30.71	0.35	-	-
	Vd (L)	117.63	87.33	1.35	-	-
	CL (L/h)	1.66	1.88	0.91	-	-
1 mg, oral solution 18	AUC (µg/L*h)	63.83	133.81	0.50	1.02	1.57
	C_max_ (µg/L)	8.68	9.99	0.87	-	-
	Vd (L)	92.92	114.77	0.81	-	-
	CL (L/h)	16.67	7.47	2.09	-	-
1 mg, oral tablet 18	AUC (µg/L*h)	150.76	149.61	1.01	1.02	1.67
	C_max_ (µg/L)	8.80	9.99	0.88	-	-
	Vd (L)	162.89	131.07	1.24	-	-
	CL (L/h)	6.63	6.68	0.99	-	-
0.6 mg, oral solution 14	AUC (µg/L*h)	38.99	19.79	1.97	0.98	2.00
	C_max_ (µg/L)	2.32	2.66	0.87	-	-
	Vd (L)	334.54	382.80	0.87	-	-
	CL (L/h)	15.38	30.31	0.51	-	-
0.5 mg, oral solution 22	AUC (µg/L*h)	19.36	20.31	0.95	1.02	1.67
	C_max_ (µg/L)	3.74	2.60	1.44	-	-
	Vd (L)	595.05	1,174.44	0.51	-	-
	CL (L/h)	25.83	24.62	1.05	-	-
1.5 mg, oral solution 22	AUC (µg/L*h)	34.15	24.08	1.42	0.96	1.57
	C_max_ (µg/L)	5.17	2.95	1.75	-	-
	Vd (L)	2,027.33	2,397.31	0.85	-	-
	CL (L/h)	43.92	62.29	0.71	-	-

AFE: Average folder error; AAFE: absolute average folder error; FE: folder error; AUC: area under the curve; C_max_: maximum concentration; Vd: volume of distribution; CL: clearance.

### Interactions between CQ and CC

The final PBPK models for CQ and CC were simulated to assess the possibility of DDIs mediated by CYP3A4 and CYP2D6. Given that both drugs are substrates of CYP3A4, they were evaluated for competitive inhibition when co-administered. In the development of new medicinal products, particularly during the preformulation phase, the ability to predict DDIs is essential to prevent costly and time-consuming failures in later developmental stages (31). Beyond ensuring safety and efficacy, early DDI modeling facilitates rational design decisions, such as the choice of excipients or the implementation of structural modifications to drug candidates (2,28). Furthermore, in the context of diseases like malaria, where treatment often involves polypharmacy and a narrow therapeutic window, anticipating interactions is not merely strategic but essential to preventing therapeutic failure or toxicity (7,10).

The ratio of the AUC and C_max_ when CQ was the affected drug (i.e., with CC acting as a competitive inhibitor) remained at 1.00 for both AUC and C_max_. Conversely, when CC was the affected drug, the ratios of AUC and C_max_ were 1.12 and 1.18, respectively. These simulations specifically aimed to evaluate the potential for CYP3A4-mediated interactions, as both drugs are substrates of this enzyme (Supplementary Figure S6). According to the International Council for Harmonisation M12 guideline on drug interaction studies (28), a DDI is not deemed clinically significant if the ratios of AUC and C_max_ between the combination therapy and monotherapy fall within the 0.80-1.25 range. Based on this criterion, the co-administration of CQ and CC did not result in a clinically relevant CYP3A4-mediated interaction, remaining within the accepted limits.

The simulation duration of 96 hours for CQ and 48 hours for CC was based on their pharmacokinetic properties, where plasma levels generally reach the elimination phase within this timeframe after a single-dose administration (14). Although standard antimalarial therapy involves multiple doses, single-dose simulations are frequently employed as an initial step to evaluate potential DDIs during early co-exposure, when competitive inhibition is the most likely to occur (10). Future studies are planned to investigate repeated-dose regimens to assess cumulative interactions that may develop over the full course of therapy (7).

Most drugs available undergo metabolism by CYP450 enzymes, resulting in interactions when drugs are co-administered (10,29). Maldonado and Grundmann (7) emphasized the necessity for *in vivo* evaluation of antimalarial DDIs due to the common metabolic pathways shared by many antimalarials. Significantly, inflammatory conditions, as observed in acute malaria, may suppress CYP3A4 activity. This suppression is supported by clinical pharmacokinetic evidence demonstrating reduced quinine CL in patients with inflammation (32) and mechanistic reviews indicating cytokine-mediated downregulation of CYP3A4 expression (33). Consequently, disease-state considerations are imperative in DDI modeling, particularly when antimalarials are administered during active infection (33,34). Given these disease-induced metabolic alterations and the limited availability of validated inflammatory PBPK models, our current simulations were concentrated on healthy physiological conditions to establish a reliable baseline for future extensions into disease-specific scenarios.

CQ and CC are metabolized primarily by CYP3A4 (35,36). Hence, CQ and CC could potentially compete for the same active site, suggesting a possible DDI (10), which was not observed in the simulations. Conversely, a PBPK model was developed to evaluate the competitive inhibition between metoprolol and tramadol on CYP2D6, given that both are substrates. The findings revealed that tramadol acted as a perpetrator, increasing metoprolol exposure by 48% (37).

When developing the PBPK model for CC, it was important to consider existing clinical evidence of pharmacokinetic interactions involving CYP3A4 and P-gp, due to their central role in CC's disposition. A clinical study showed that CC interacted with CYP3A4/P-gp inhibitor drugs, demonstrating a dose adjustment of CC when co-administered with potent dual inhibitors of CYP3A4 and P-gp (e.g., verapamil and diltiazem), whereas no dose adjustment was required with azithromycin (14). These data informed the selection of input parameters and sensitivity scenarios in our PBPK simulations, particularly for exploring potential DDI mechanisms at absorption and hepatic metabolism levels.

CQ is an inhibitor of CYP2D6 both *in vitro* and *in vivo*; therefore, caution is advised when it is co-administered with drugs that are substrates of this enzyme, such as metoprolol, codeine, or risperidone (38). This inhibitory effect holds particular relevance when combined with drugs that depend on CYP2D6 for bioactivation or detoxification, as inhibition may lead to reduced efficacy or increased toxicity (39). CC is a substrate of CYP2D6 (40); nevertheless, our PBPK simulation revealed no modification in its exposure (AUC 1.00, C_max_ 1.00). This outcome is consistent with the relatively minor role of CYP2D6 in CC clearance, estimated to be approximately 25% (15), minimizing the risk of a clinically significant DDI (Supplementary Figure S6). The PBPK model thus provided a valuable tool for safely and mechanistically assessing this potential interaction (1). However, as with other simulations in this study, these findings are based on healthy individuals and may not fully reflect the metabolic alterations induced by malaria-associated inflammation (33,34).

Furthermore, integrating data-driven modeling strategies with PBPK and parameter optimization increases the robustness and translational potential of such models (1). These approaches enable early mechanistic screening of complex drug combinations in neglected diseases, such as malaria, where ethical and practical constraints often limit *in vivo* experimentation (16).

Although the PBPK models developed in this study provide mechanistic insight into the potential DDIs between CQ and CC, certain limitations must be acknowledged. Firstly, the simulations were conducted using physiological parameters from healthy individuals, which do not fully represent the complex pathophysiological conditions prevalent during an acute malaria infection. Inflammatory responses associated with malaria have been shown to modulate drug-metabolizing enzymes, such as CYP3A4 and CYP2D6 (33,34), potentially altering drug clearance and interaction profiles. Clinical evidence indicates that the pharmacokinetics of CQ are relatively stable between healthy volunteers and malaria patients, with comparable clearance and distribution parameters (30). In contrast, CC disposition is more susceptible to physiological changes, particularly due to its reliance on CYP3A4 metabolism and P-glycoprotein transport; inflammatory states may downregulate these pathways, potentially increasing systemic exposure and toxicity risk (33,34). Secondly, we evaluated only single-dose regimens, which may not accurately represent cumulative or time-dependent interactions arising from repeated administrations, as commonly employed in antimalarial therapy; nonetheless, both drugs exhibit linear pharmacokinetics. Thirdly, despite incorporating *in vitro* data on enzyme inhibition and metabolism, the predictive accuracy of these parameters might vary, depending on the experimental systems and scaling approaches used (10).

## Conclusions

This study demonstrated that physiologically-based pharmacokinetic modeling serves as an invaluable tool for predicting DDI during the preformulation stage. By identifying the absence of clinically significant interactions between CQ and CC early in the development process, it is possible to mitigate unnecessary patient risks and reduce costs associated with preclinical and clinical testing.

The confirmation that no DDI occurs during the co-administration of CQ and CC underlines the potential for using this combination in therapeutic regimens. Considering that our previous research validated the feasibility of their co-encapsulation into nanocapsules and confirmed their low toxicity in rats (11), advancing to efficacy studies and further translational research is the next logical step.

Moreover, integrating data-driven approaches and parameter optimization into PBPK modeling further strengthens its application in early-phase DDI risk assessment. This is particularly relevant in the rational design of novel therapeutic combinations for treating malaria.

## Data Availability

The datasets generated and/or analyzed during the current study are available from the corresponding author on reasonable request.

## Supplementary Materials

Supplementary Material Click here to view [pdf].
