# Supplementary material for: A clinical drug-drug interaction assessment using physiologically based pharmacokinetic modeling: a case study with chloroquine and colchicine
Source: Braz J Med Biol Res. 2026 Jul 10;59:e15326. doi: 10.1590/1414-431X2026e15326 (PMC13362891; doi:10.1590/1414-431X2026e15326)

Figure S1. Concentration-time profiles for chloroquine (CQ) and colchicine (CC) after an intravenous single dose in healthy subjects and a virtual population (n=100). **A**, individual CC; **B**, individual CQ; **C**, population CC; **D**, population CQ. The solid lines represent the predicted mean concentration-time profile, and the gray shaded area represents the geometric standard deviation. The dots represent clinical data observed from the study of Gustafsson et al. (19) for CQ (300 mg) and Ferron et al. (18) for CC (0.5 mg).

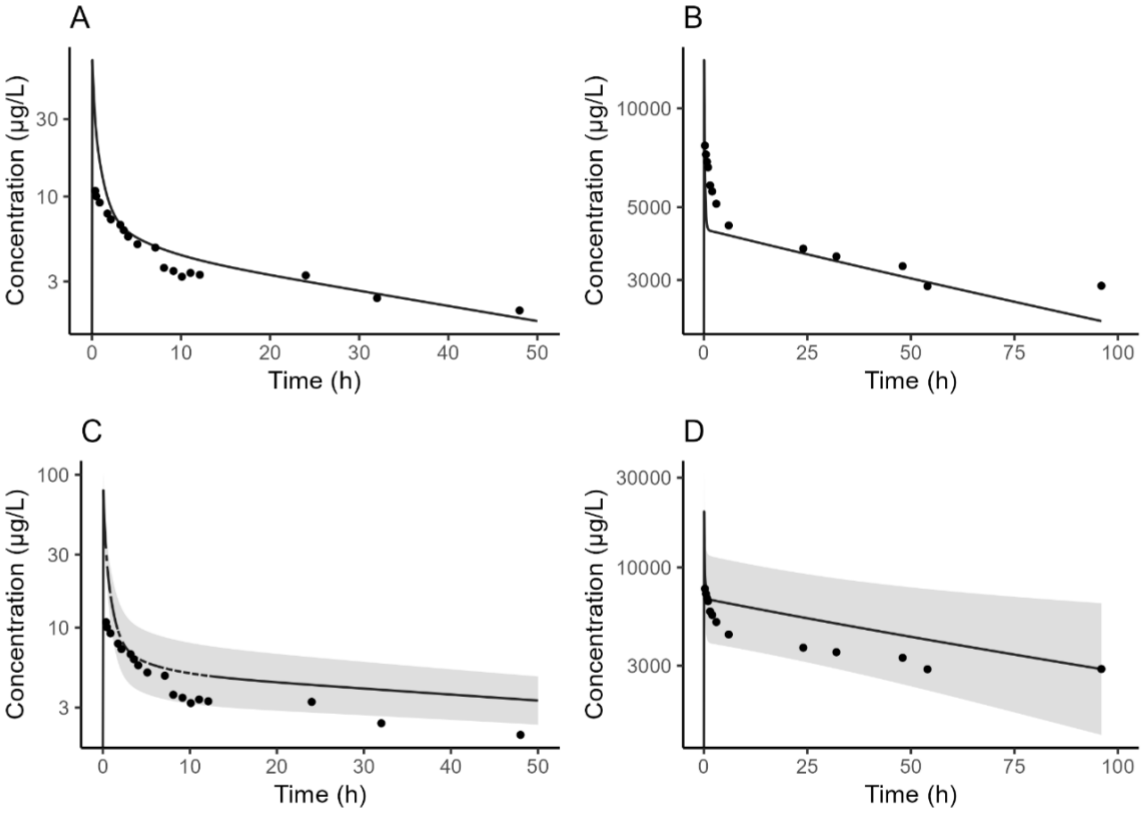

Figure S2. Concentration-time profiles for colchicine (CC) after a single oral dose in healthy subjects and a virtual population (n=100). **A**, individual CC (1 mg, oral solution); **B**, population CC (1 mg, oral solution); **C**, individual CC (1 mg, tablet); **D**, population CC (1 mg, tablet). The solid lines represent the predicted mean concentration-time profile, and the gray shaded area represents the geometric standard deviation. The dots represent clinical data observed in the study by Ferron et al. (18).

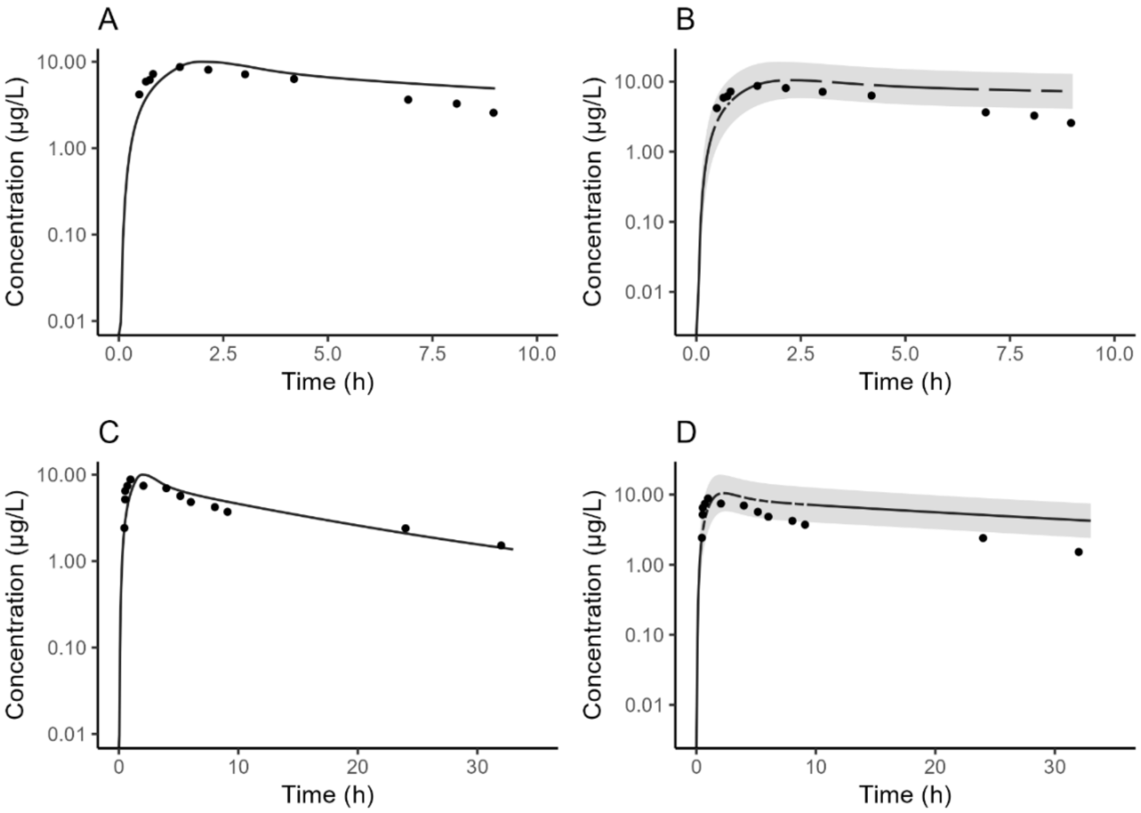

Figure S3. Concentration-time profiles for colchicine (CC) after a single oral dose in healthy subjects and a virtual population (n=100). **A**, individual CC (Terkeltaub et al. (14)); **B**, population CC (0.6 mg, Terkeltaub et al. (14)); **C**, individual CC (Thomas et al. (22)); **D**, population CC (1.5 mg, Thomas et al. (22)). The solid lines represent the predicted mean concentration-time profile, and the gray shaded area represents the geometric standard deviation. The dots represent clinical data observed in the studies.

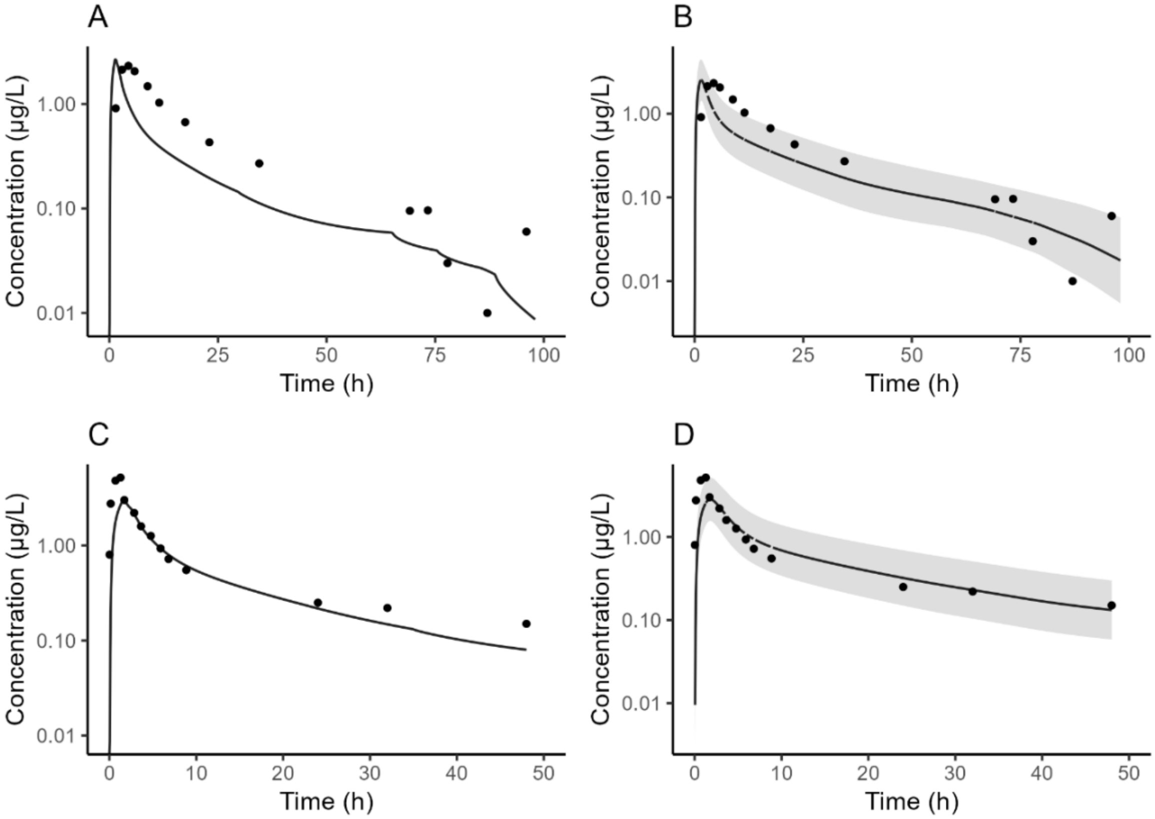

Figure S4. Concentration-time profiles for chloroquine (CQ) after a single oral dose in healthy subjects and a virtual population (n=100). **A**, individual CQ (300 mg tablet, Gustafsson et al. (19)); **B**, population CQ (300 mg tablet, Gustafsson et al. (19)); **C**, individual CQ (500 mg, Neuvonen et al. (20)); **D**, population CQ (500 mg, Neuvonen et al. (20)). The solid lines represent the predicted mean concentration-time profile, and the gray shaded area represents the geometric standard deviation. The dots represent the clinical data observed in the studies.

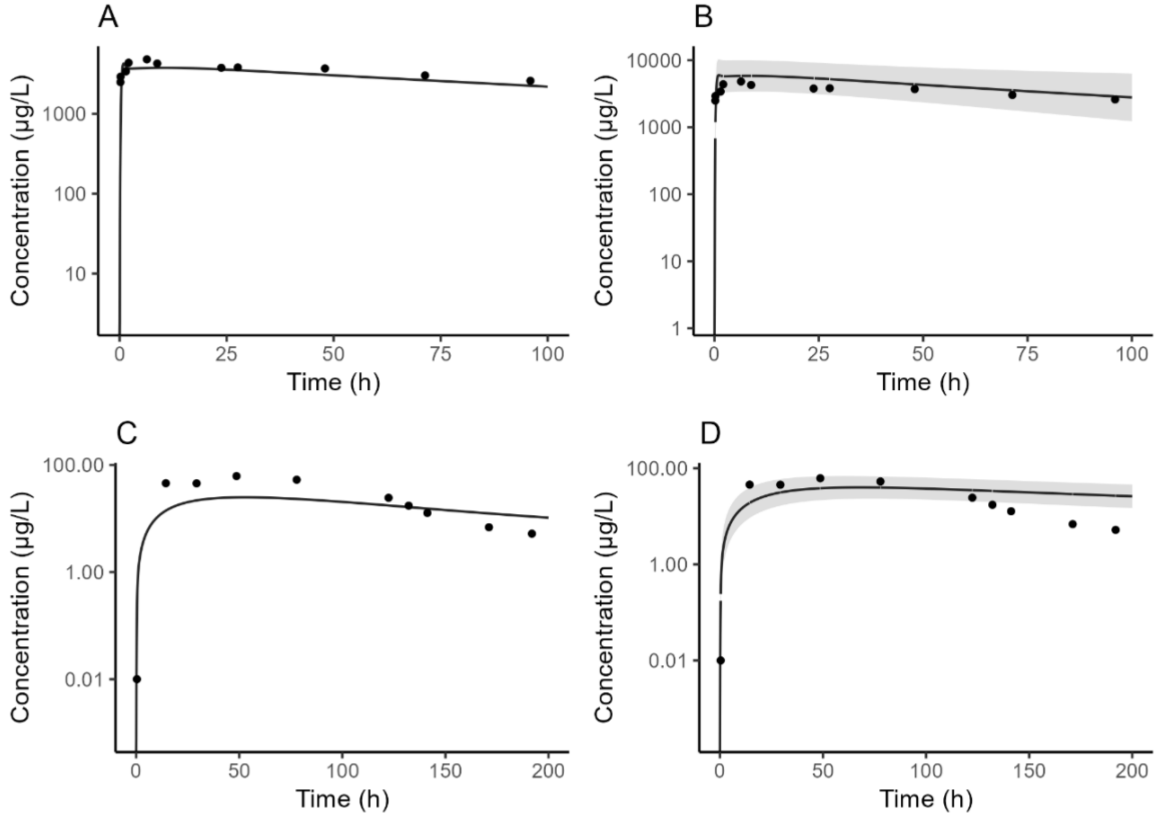

Figure S5. Concentration-time profiles for chloroquine (CQ) after a single oral dose in healthy subjects and a virtual population (n=100). **A**, individual CQ (600 mg, Pukrittayakamee et al. (21)); **B**, population CQ (600 mg, Pukrittayakamee et al. (21)); **C**, individual CQ (600 mg, Walker et al. (23)); **D**, population CQ (600 mg, Walker et al. (23)). The solid lines represent the predicted mean concentration-time profile, and the gray shaded area represents the geometric standard deviation. The dots represent the clinical data observed in the studies.

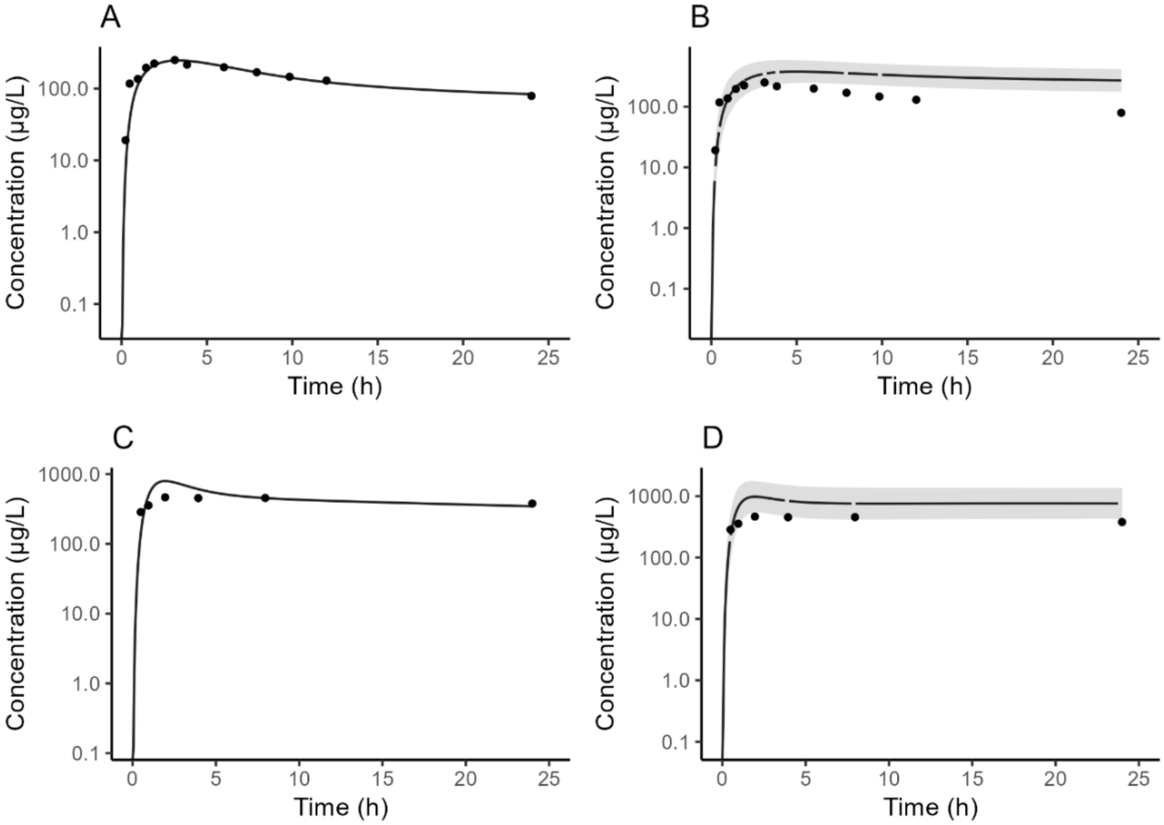

Figure S6. Concentration-time profiles for a single oral dose in healthy subjects after drug-drug interactions assessment. **A**, chloroquine (CQ) victim (CYP3A4); **B**, colchicine (CC) victim (CYP3A4); **C**, CC victim (CYP2D6). The solid lines represent the predicted mean concentration-time profile. The dots represent the clinical data observed in the studies by Gustafsson et al. (19) for CQ and Thomas et al. (22) for CC.

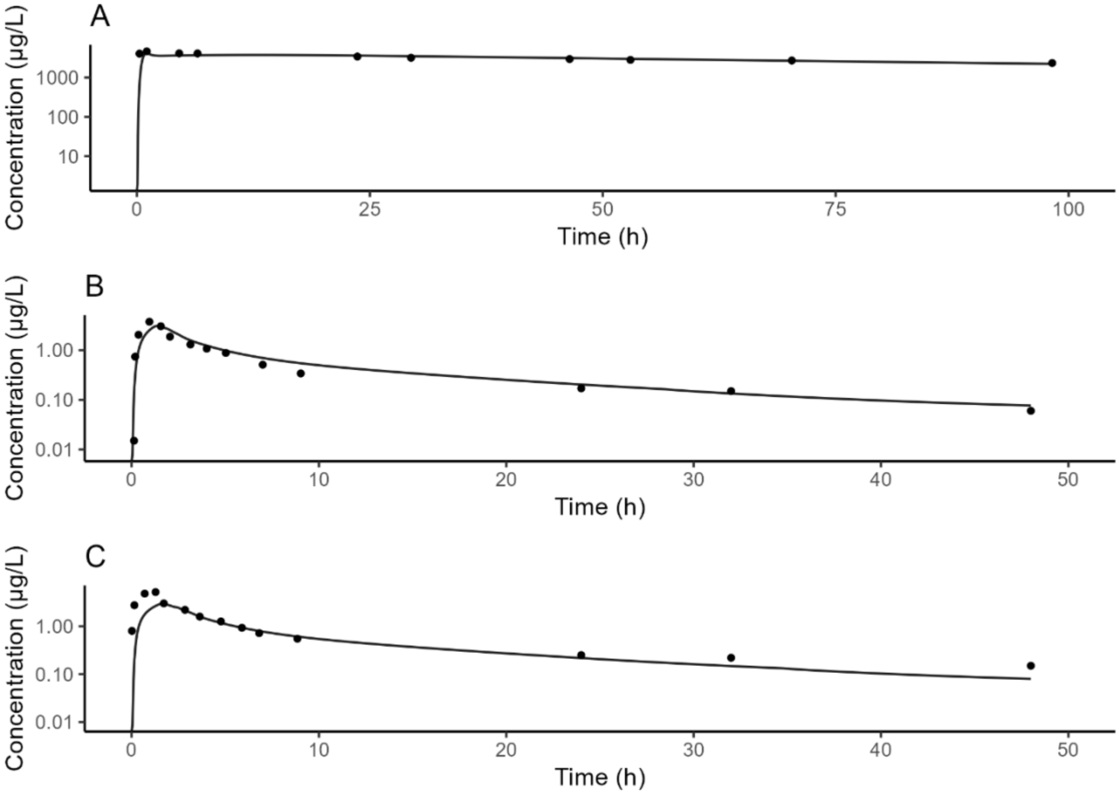

Supplement: Supplementary Material [file 1414-431X-bjmbr-59-e15326-suppl.pdf]
